# Analysis of risk factors related to extremely and very preterm birth: a retrospective study

**DOI:** 10.1186/s12884-022-05119-7

**Published:** 2022-11-05

**Authors:** Xiaohong Ji, Chengqian Wu, Min Chen, Lili Wu, Ting Li, Zhijing Miao, Yan Lv, Hongjuan Ding

**Affiliations:** Women’s Hospital of Nanjing Medical University (Nanjing Maternity and Child Health Care Hospital), 210004 Nanjing, China

**Keywords:** Extremely preterm birth, Very preterm birth, Risk factor, Multivariate

## Abstract

**Background:**

Preterm birth is one of the main causes of perinatal morbidity and mortality and imposes a heavy burden on families and society. The aim of this study was to identify risk factors and analyze birth conditions and complications of newborns born at < 32 gestational weeks for extremely preterm (EP) and very preterm (VP) birth in the clinic to further extend the gestational period.

**Methods:**

We performed a retrospective cohort study and collected data from 1598 pregnant women and 1660 premature newborns (excluding 229 premature babies who died due to severe illness and abandonment) admitted to the Obstetrics and Gynecology Hospital Affiliated with Nanjing Medical University in China from 2016 to 2020. We compared women’s and newborns’ characteristics by t-tests and Chi-square tests for continuous and categorical variables, respectively. Multivariable logistic regression was performed to estimate the effects of risk factors on EP and VP birth.

**Results:**

We identified 3 independent risk factors for EP birth: cervical incompetency (*P* < 0.001); multiple pregnancy (*P* < 0.01), primipara (*P* < 0.001). Additionally, we identified 4 independent risk factors for VP birth: gestational diabetes mellitus (GDM) (*P* < 0.05), preterm premature rupture of membrane (PPROM) (*P* < 0.01), fetal intrauterine distress (*P* < 0.001), and hypertensive disorder complicating pregnancy (HDCP) (*P* < 0.001). In addition, pairwise comparisons revealed statistically significant differences in the incidence rates of neonatal pneumonia, bronchopulmonary dysplasia (BPD) and sepsis between the 28–28 + 6 and 29–29 + 6 weeks of gestation groups (*P* < 0.05). Compared with 28–28 + 6 weeks of gestation, neonatal complications were significantly more common at < 26 weeks of gestation (*P* < 0.05). The incidence rates of neonatal intracranial hemorrhage(NICH), patent ductus arteriosus(PDA), patent foramen ovale(PFO), pneumonia, BPD and sepsis were significantly higher in the 26–26 + 6 and 27–27 + 6 gestational weeks than in the 28–28 + 6 gestational weeks (*P* < 0.05).

**Conclusion:**

PPROM, is the most common risk factor for EP and VP birth, and cervical insufficiency, multiple pregnancy, and primipara are independent risk factors for EP birth. Therefore, during pregnancy, attention should be devoted to the risk factors for PPROM, and reproductive tract infection should be actively prevented to reduce the occurrence of PPROM. Identifying the risk factors for cervical insufficiency, actively intervening before pregnancy, and cervical cervix ligation may be considered to reduce the occurrence of EP labor. For iatrogenic preterm birth, the advantages and disadvantages should be carefully weighed, and the gestational period should be extended beyond 28 weeks to enhance the safety of the mother and child and to improve the outcomes of preterm birth.

**Supplementary Information:**

The online version contains supplementary material available at 10.1186/s12884-022-05119-7.

## Introduction

Preterm birth, especially extremely preterm (EP) birth and very preterm (VP) birth, is a major challenge for newborns and their families as well as for perinatologists and neonatologists [1]. The World Health Organization defines preterm birth as birth occurring before 37 weeks of gestation; the subcategories of preterm birth include EP birth (< 28 weeks of gestation), VP birth (28 to 31 + 6 weeks of gestation), and moderate-to-late preterm birth (32 to 36 + 6 weeks of gestation) [2]. There is heterogeneity in the preterm birth rate reported in different studies [3, 4, 5]. Among the 15 million preterm births that occur annually worldwide, approximately 5% are EP births, and 10% are born at 28–31 weeks of gestation [6]. A multi-center survey involving 89 hospitals in 25 provinces in China showed that the incidence of preterm birth was 7.3% between 2015 and 2016 [4]. While as reported in the World Health Organization (WHO) Report in 2019, the global had 14.8 million premature babies with the average preterm birth rate of 10.6%, and China had more than 1.1 million PTBs with the rate of 6.9% [5]. With the development of perinatal care and technology, survival among infants born at < 32 gestational weeks has improved dramatically over the past several decades [7, 8]. However, the prevalence of adverse medical and neurodevelopmental outcomes for those born at < 32 gestational weeks remain high [9, 10, 11], particularly for infants born at < 28 gestational weeks.

In recent years, the rate of preterm birth has increased in many counties [12]. Preterm labor is now thought to be a complex process initiated by multiple mechanisms. Causes [12, 13] of preterm birth occurring at less than 32 weeks of gestation have been hierarchically classified as infection or inflammation, preterm premature rupture of membrane (PPROM) [14], pregnancy hemorrhage (i.e., placental abruption and placenta previa), hypertensive diseases (essential hypertension, gestational hypertension, and preeclampsia), intrauterine growth retardation, and other immunologically mediated processes. Many studies [8, 15, 16] have shown that infants born at < 32 gestational weeks accounted for 12.9% of preterm births, > 50% of death and neurodevelopmental disabilities happen to them. Herein, birth conditions and complications of newborns born at < 32 gestational weeks were analyzed to verify this conclusion, and to compare neonatal complications at each gestational week to provide a basis for extension to a specific gestational week. In addition, we analyzed the factors related to EP and VP birth and determined the differences in related factors so that we could identify high-risk factors in the clinic to further extend the gestational period.

## Materials and methods

### Study subjects

**Fig. 1:**
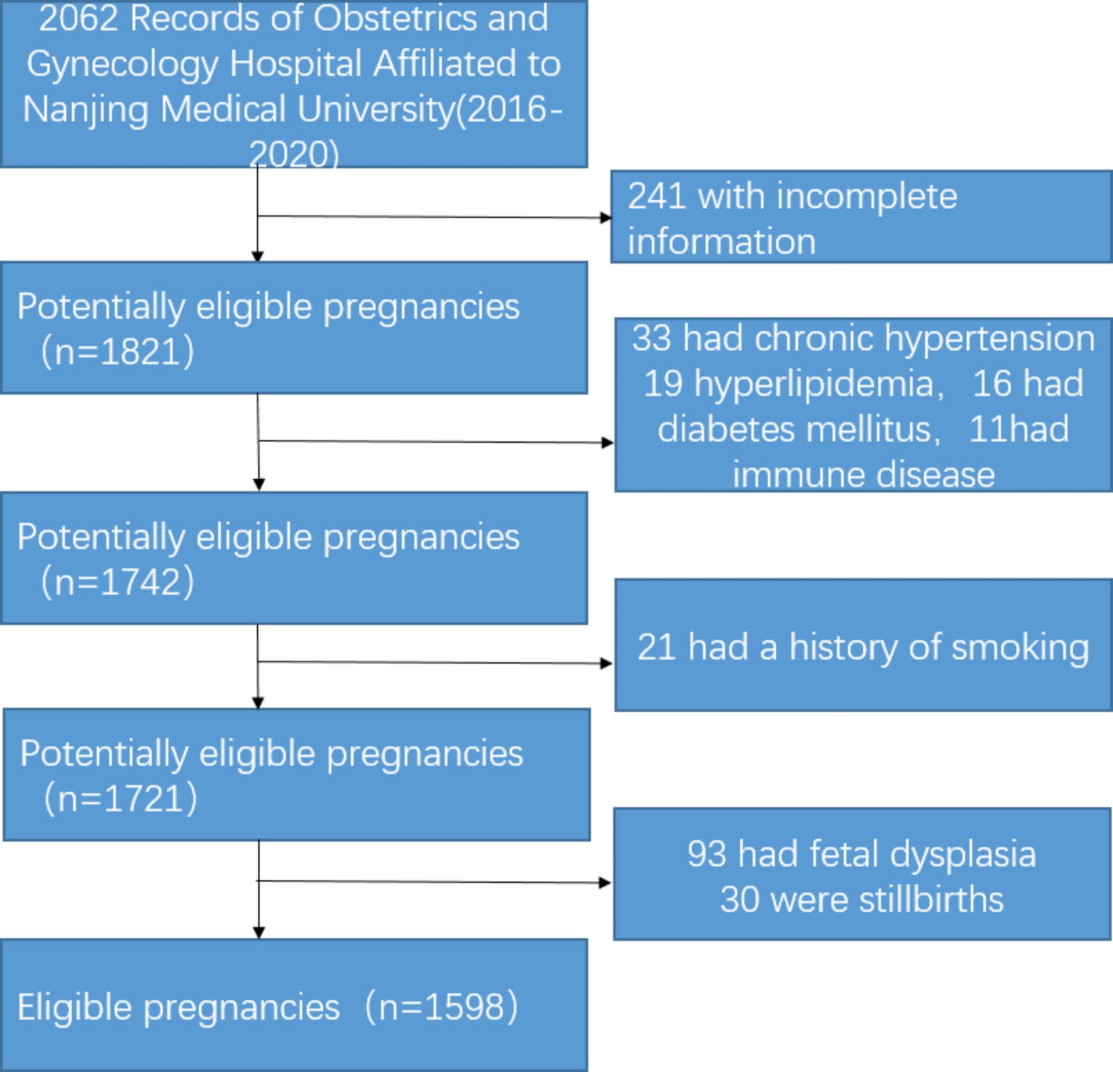
Flowchart for inclusions in the study

Our primary hypothesis was that there would be a difference in factors related to EP and VP birth. We performed a retrospective cohort study and collected data from 2062 pregnant women admitted to Obstetrics and Gynecology Hospital Affiliated with Nanjing Medical University in China from 2016 to 2020. Gestational age was determined based on the last menstruation and the earliest available reliable ultrasound data; if the last menstruation was unknown or uncertain, gestational age was determined using standard ultrasound data. A total of 241 women with incomplete information were excluded. We excluded 79 women with chronic diseases (chronic hypertension, hyperlipidemia, diabetes mellitus) and immune diseases. We also excluded 21 women who had a history of smoking. A total of 123 women who experienced abortions, induced labor, or stillbirths were also excluded in Fig. 1. A total of 1889 babies were born at less than 32 weeks of gestation, including 229 premature babies who died due to severe illness and abandonment.

### Ethical approval

The study followed the Declaration of Helsinki and was conducted in accordance with the relevant local guidelines and regulations. The study was approved by theMedical Ethics Committee of Nanjing Maternal and Child Health Hospital, and informed consent was waived (No. 2022KY-033, Supplementary Material 1).

### Statistical analysis

In this study, we compared women’s and newborns’ characteristics by t-tests and Chi-square tests for continuous and categorical variables, respectively. Multivariable logistic regression was performed to estimate the effects of risk factors on EP and VP birth, i.e., scarred uterus, preterm premature rupture of membranes (PPROM), multiple pregnancy, cervical incompetency, hypertensive disorder complicating pregnancy (HDCP), gestational diabetes mellitus (GDM), and abnormal fetal position. Risk factors for EP birth were identified by using binominal logistic regression models. We included scarred uterus, PPROM, multiple pregnancy, cervical incompetency, HDCP, GDM, and abnormal fetal position in the models. Odds ratios (ORs) were used to estimate relative risk with 95% confidence intervals (CIs). Adjusted ORs and 95%CIs were calculated to present the risk. All analyses were conducted using R software (version 3.2.4, R Foundation for Statistical Computing, Vienna, Austria). We also retrospectively analyzed the complications, birth weight, apgar score 1–5 min after birth, rate of abandonment during treatment, rate of recovery and hospital stay in the neonatal intensive care unit (NICU) of infants born at < 32 gestational weeks. Categorical variables are presented as frequencies, and continuous variables are expressed as the mean (standard deviation). *P* < 0.05 was considered significant (^*^P < 0.05, ^**^P < 0.01, ^***^P < 0.001).

## Results

### Comparison of general risk factors in pregnant women with EP and VP births

Furthermore, we analyzed the risk factors for EP and VP births. The study cohort consisted of 1598 pregnant women. Demographic and clinical characteristics for EP (n = 287) and VP (n = 1311) births are shown in Table 1. There were no significant differences between the 2 groups in the following risk factors such as maternal age, miscarriages ≥ 3, primipara, days admitted to the hospital, blood type, abnormal placenta and sex of the newborn (*P* > 0.05). The probability of EP and VP births differed in pregnant women with the following risk factors: scarred uterus, PPROM, GDM, multiple pregnancy, abnormal fetal position, cervical insufficiency, fetal intrauterine distress, hypertensive disease during pregnancy, abnormal amniotic fluid, abnormal umbilical cord blood flow, and chorioamnionitis (*P <* 0.05).

**Table 1:** Comparison between women with EP and VP births. BMI, body mass index. *, t value

Characteristics	Number(n)	EP(n = 287)	VP(n = 1311)	χ^2^/t	*P*
Age (yrs), mean (SD)	1598 (100.00)	30.36 (3.85)	30.40 (4.01)	0.60^*^	0.437
Abortion ≥ 3, n (%)	133 (8.32)	29 (10.10)	104 (7.93)	1.46	0.228
Primipara, n (%)	948 (59.32)	201 (70.03)	747 (56.98)	16.63	0.000
BMI ≥ 25, n (%)	680 (42.55)	112 (39.02)	568 (43.33)	1.78	0.182
Days admitted to the hospital ≥ 3, n (%)	275 (17.21)	50 (17.42)	225 (17.16)	0.01	0.916
Assisted reproduction, n (%)	272 (17.02)	51 (17.77)	221 (16.86)	0.14	0.709
Blood type of O, n (%)	552 (34.54)	92 (32.06)	460 (35.09)	0.96	0.328
Scar uterus, n (%)	304 (19.02)	35 (12.20)	269 (20.52)	10.59	0.001
Male newborn, n (%)	908 (56.82)	173 (60.28)	735 (56.06)	1.70	0.192
PPROM, n (%)	716 (44.81)	103 (35.89)	613 (46.76)	11.25	0.001
GDM, n (%)	354 (22.15)	50 (17.42)	304 (23.19)	4.54	0.033
Multiple pregnancy, n (%)	289 (18.09)	71 (24.74)	218 (16.63)	10.45	0.001
Abnormal fetal position, n (%)	331 (20.71)	45 (15.68)	286 (21.82)	5.40	0.020
Placental factors, n (%)	167 (10.45)	22 (7.67)	145 (11.06)	2.90	0.089
Cervical incompetency,n (%),	112 (7.01)	40 (13.94)	72 (5.49)	25.77	0.000
Fetal distress, n (%)	178 (11.14)	6 (2.09)	172 (13.12)	28.94	0.000
HDCP, n (%)	185 (11.58)	7 (2.44)	178 (13.58)	28.54	0.000
Amniotic fluid anomaly, n (%)	287 (17.96)	36 (12.54)	251 (19.15)	6.97	0.008
Abnormal vasa umbilicalis, n (%)	114 (7.13)	8 (2.79)	106 (8.09)	9.98	0.002
Chorioamnionitis, n (%)	360 (22.53)	54 (18.82)	306 (23.34)	2.76	0.096

### Univariate and multivariate logistic regression

**Fig. 2:**
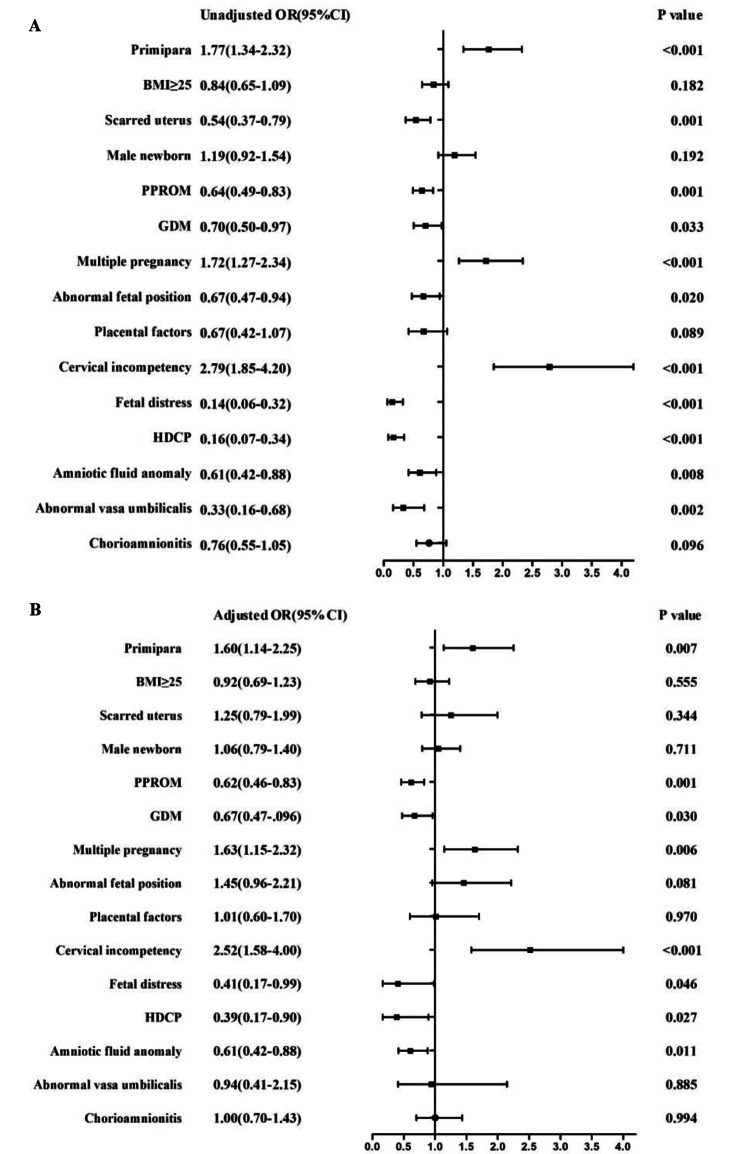
**A.** Unadjusted odds ratios of clinical characteristics and other diseases in Table 1 for EP and VP births. **B.** Adjusted odds ratios of clinical characteristics and other diseases in Table 1 for EP and VP births, using multivariable logistic regression

Since EP and VP births are highly complex processes, they are influenced by multiple factors. The variables in table 1 with *P* < 0.20 in the single-factor analysis (Fig. 2A) were subjected to multivariate logistic regression analysis (Fig. 2B), which we carried out to explore independent risk factors for EP and VP birth. The results are shown in Fig. 2 A-B. Following adjustment for some variables listed in Table 1, we identified 3 independent risk factors for EP birth: cervical incompetency (unadjusted OR 2.79, 95%CI 1.85–4.20; adjusted OR 2.52, 95%CI 1.58-4.00), multiple pregnancy (unadjusted OR 1.72, 95%CI 1.27–2.34; adjusted OR 1.63, 95%CI 1.15–2.32), and primipara (unadjusted OR 1.77, 95%CI 1.34–2.32; adjusted OR 1.60, 95%CI 1.14–2.25).We also identified 4 independent risk factors for VP birth: GDM (unadjusted OR 0.70, 95%CI 0.50–0.97; adjusted OR 0.67, 95%CI 0.47–0.96), PPROM (unadjusted OR 0.64, 95%CI 0.49–0.83; adjusted OR 0.62, 95%CI 0.46–0.83), fetal intrauterine distress (unadjusted OR 0.14, 95%CI 0.06–0.32; adjusted OR 0.41, 95%CI 0.17–0.99), and HDCP (unadjusted OR 0.16, 95%CI 0.07–0.34; adjusted OR 0.39, 95%CI 0.17–0.90).

### Comparison of complications among neonates born at less than 32 weeks of gestation

**Fig. 3:**
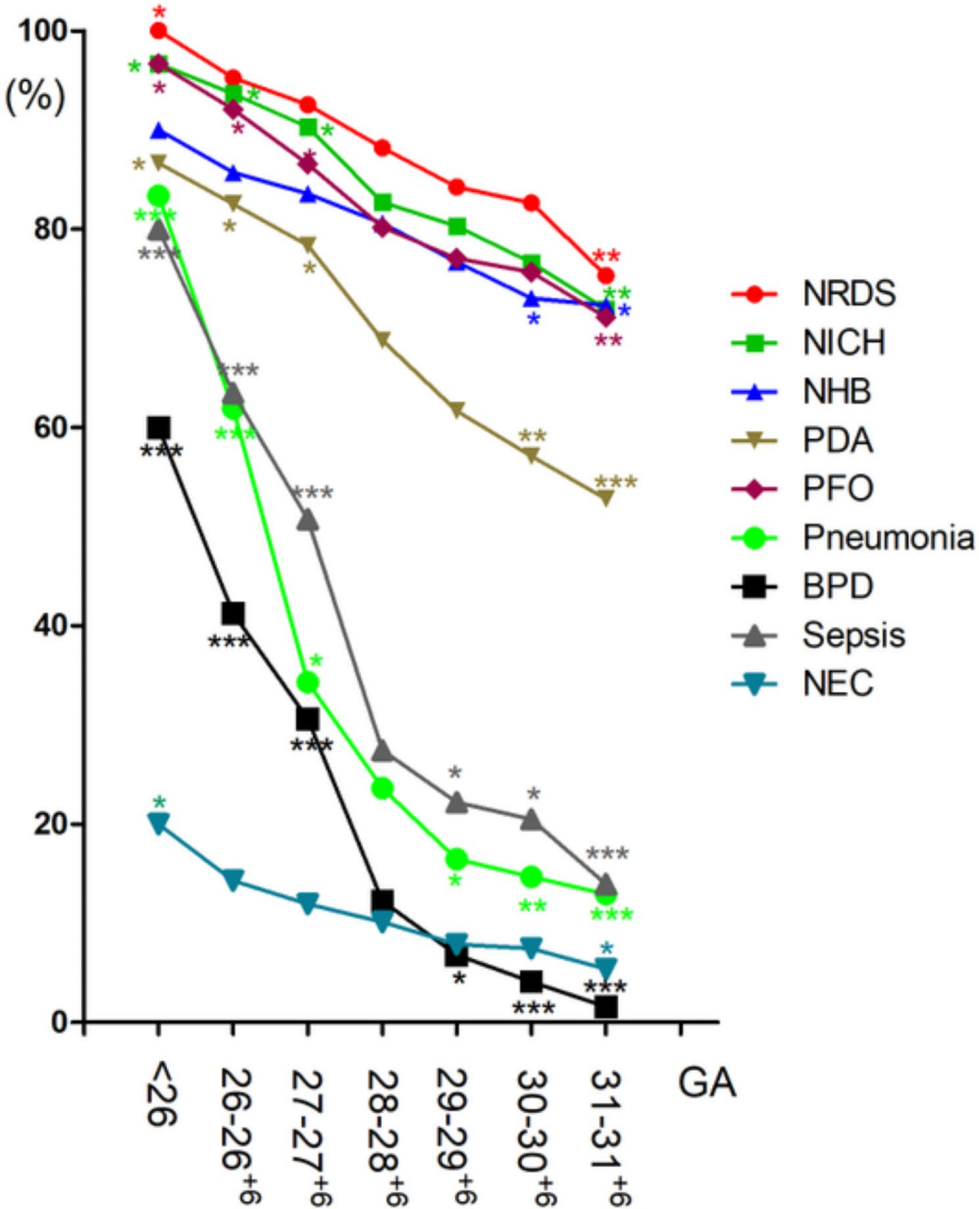
Comparison of complications of premature infants at different gestational ages. The control group was 28–28 + 6 weeks of gestation. NRDS, neonatal respiratory distress syndrome. NICH, neonatal intracranial hemorrhage. NHB, neonatal hyperbilirubinemia. PDA, patent ductus arteriosus. PFO, patent foramen ovale. BPD, bronchopulmonary dysplasia. NEC, neonatal necrotizing enterocolitis

We excluded 229 premature babies who died due to severe illness and abandonment during the treatment, as shown in Fig. 3 (about 1660 babies after excluded 229 babies who died). The prevalence of complications associated with premature birth is related to gestational age and decreases with gestational age. Pairwise comparison revealed significant differences in the incidence of neonatal pneumonia, bronchopulmonary dysplasia (BPD) and sepsis between the 28–28 + 6 and 29–29 + 6 weeks of gestation groups (*P* < 0.05), while other complications, including neonatal respiratory distress syndrome (NRDS), neonatal intracranial hemorrhage (NICH), neonatal hyperbilirubinemia (NHB), patent ductus arteriosus (PDA), patent foramen ovale (PFO), sepsis, and neonatal necrotizing enterocolitis (NEC), did not differ significantly (*P* > 0.05). Compared with the 28–28 + 6 weeks of gestation group, neonatal complications were more common among the < 26 weeks of gestation group (*P* < 0.05). There was no significant difference in the incidence of complications such as NRDS, NHB and NEC between newborns born at 26–26 + 6 and 27–27 + 6 weeks of gestation and those born at 28–28 + 6 weeks of gestation (*P* > 0.05). The incidence rates of NICH, PDA, PFO, pneumonia, BPD and sepsis were significantly higher among newborns born at 26–26 + 6 and 27–27 + 6 weeks of gestation than among those born at 28–28 + 6 gestational weeks (*P* < 0.05). The incidence rates of complications such as NRDS, NICH, PFO and NEC were not significantly different between neonates born at 30–30 + 6 gestation and those born at 28–28 + 6 gestation (*P* > 0.05). The incidence of NHB, PDA, pneumonia, BPD and septicemia were significantly lower among neonates born at 30–30 + 6 gestation weeks than among those born at 28–28 + 6 gestational weeks (*P* < 0.05). Compared with newborns at 28–28 + 6 gestation weeks, the rate of neonatal complications significantly decreased at 31–31 + 6 weeks of gestation (*P* < 0.05).

### Comparison of birth outcomes between EP and VP neonates

As shown in Fig. 3, births occurring at less than 32 weeks’ gestation were divided into two groups, namely, EP and VP births. The neonatal survival and mortality rates in the VP and EP groups are shown in Table 2. Among the 1,889 premature births, 358 were EP births and 1531 were VP births. The cure rate and recovery rate of neonates in the EP group were significantly lower than those in the VP birth group (*P* < 0.01), while the rates of hospital transfer, severe disease and mortality were significantly higher than those in the VP infant group (*P* < 0.001).

Then, we analyzed the birth status of surviving preterm infants, including 266 EP infants and 1394 VP infants (Table 3). The birth weight, Apgar 1 min and Apgar 5 min of EP infants were significantly lower than those of VP infants, while the number of hospitalization days in NICU were significantly higher than those of VP infants. There was no statistical difference (*P* > 0.05) in discharge weight of infants between the two groups.

**Table 2:** Survival and mortality rates in EP and VP birth groups

	Survival			Mortality	
	Cure	Mend	Transfer	Diseaseseverity	Abandonment
EP, n(%)	220 (61.45)	32 (8.94)	14 (3.91)	25 (6.98)	67 (18.72)
VP, n(%)	1151 (75.18)	228 (14.89)	15 (0.98)	32 (2.09)	105 (6.86)
χ2	27.471	8.665	16.488	23.739	49.287
*P*	< 0.001	0.003	< 0.001	< 0.001	< 0.001

**Table 3:** Birth status and hospitalization in the surviving EP and VP birth groups

	EP (n = 266)	VP (n = 1394)	χ^2^/t	*P*
Birth weight (g), mean (SD)	1062.34 (182.97)	1518.62 (300.36)	33.003	0.000
Apgar score at 1 min, mean (SD)	7.68 (2.14)	9.14 (1.54)	10.632	0.000
Apgar score at 5 min, mean (SD)	8.89 (1.28)	9.66 (0.84)	9.457	0.000
Days in NICU (d), mean (SD)	51.72 (16.90)	27.72 (48.42)	-7.989	0.000
Weight at discharge (g), mean (SD)	1897.84 (373.53)	1874.87 (237.81)	-0.962	0.337

## Discussion

PPROM [17] is one of the leading causes of premature birth, accounting for 30–40% of all preterm births. Consistently, in this study, PPROM was the primary risk factor for both EP and VP births (36.28% vs. 46.76%). In addition, we identified 3 independent factors for EP births and 4 independent risk factors for VP birth. A previous study showed that cervical insufficiency is an important cause of recurrence of second trimester miscarriage and EP delivery [18, 19], which was consistent with our findings. As an independent risk factor, our data show that the odds of EP birth among women with cervical incompetency were 2.515 times greater (95% CI 1.58-4.00) than those of VP women. For pregnant women with cervical insufficiency, prophylactic cervical cerclage could significantly reduce the incidence of EP birth [18]. Krupa et al. reported [20] that fewer than 1% of singleton pregnancies result in EP birth, while 5% of twins are delivered before 28 weeks gestation and 12.1% of twins are delivered before 32 weeks. As shown in Fig. 3, compared to VP birth, multiple pregnancy was the second-strongest independent risk factor (adjusted OR 1.63, 95%CI 1.15–2.32) for EP birth. With EP infants being at higher risk of poor outcomes, including death, twins are five times and triplets nearly 15 times more likely than singletons to die within one month of birth [21]. Interestingly, we found that primiparas had a 1.60-fold (95%CI 1.14–2.25) increased risk of EP birth. Consistently, Li et al. [22] analyzed the risk factors among 139 premature infants born in Shenzhen from 2003 to 2012 and revealed that the probability of preterm birth in primiparas was higher than that in multiparas. The association between parity and EP birth needs further investigation.

Surprisingly, GDM, PROM, fetal distress, and HDCP were protective factors for EP birth compared with VP birth, with adjusted odds ratios of 0.67, 0.62, 0.41, and 0.39, respectively. This finding is likely related to the mechanism and timing of these diseases.

The comparison with extremely preterm and very preterm births, which have poorer neonatal outcomes compared to full-term births [23, 24], may be less informative for clinical work. In a follow-up study, we will match a certain number of pregnant women delivered at term to compare the difference in prognosis between extremely preterm and very preterm newborns and full-term newborns. Each year, more than 60,000 neonates are born at less than 32 weeks of gestation [25]. In this study, the minimum birth weight of cured premature infants was 700 g at 25 + 4weeks gestational age, and these neonates were hospitalized in the NICU for 63 days before being discharged. Our data suggested that the birth weight and Apgar1-5 min score of EP infants were significantly lower than those of VP infants (*P* < 0.001), which were similar to those of a previous study [8], and hospitalization days in NICU and discharge weight of EP infants were significantly higher than those of VP infants (*P* < 0.001). Premature births due to an immature organ system are prone to many complications. A decrease in gestational age has been shown to be strongly associated with mortality and moderately associated with major neonatal morbidity [23]. In our study, there were significant differences in the incidence rates of neonatal pneumonia, BPD and sepsis between the 28–28 + 6 weeks of gestation and the 29–29 + 6 weeks of gestation groups, while other complications, including NRDS, NICH, NHB, PDA, PFO, sepsis, and neonatal NEC, did not differ significantly between these groups. This may provide a basis for extending the gestational period beyond 28 weeks when EP birth is unavoidable while ensuring the safety of the mother and child.

## Limitations

Although we included many factors, there were some factors that may be missing or ignored due to not recorded in the medical record. For example, we did not consider smoking as a risk factor [12], these data are not fully available in our medical records. The 21 smokers in our inclusion flow chart are exact, and there are other uncertainties, and the smoking rate among Chinese women is low [26], so it is unlikely that our results will be meaningfully biased. In addition, our study was single-center and retrospective in nature. A multi-center study would be more meaningful and convincing, and it may be necessary to conduct multi-center research for the follow-up study.

## Conclusion

PPROM is the most common risk factor for EP and VP births, and cervical insufficiency/multiple pregnancy/primipara is an independent risk factor for EP birth. The morbidity and mortality of premature infants are closely related to gestational age. The mortality and mortality of infants born at < 28 weeks of gestation were significantly higher. Therefore, during pregnancy, attention should be given to the risk factors for PPROM, and reproductive tract infection should be actively prevented to reduce the occurrence of PPROM. Identifying the risk factors for cervical insufficiency, actively intervening before pregnancy, and cervical cervix ligation may be considered to reduce the occurrence of EP birth. For iatrogenic preterm birth, the advantages and disadvantages should be carefully weighed, and the gestational period should be extended beyond 28 weeks to enhance the safety of the mother and child and to improve the outcomes of preterm birth.

## Electronic supplementary material

Below is the link to the electronic supplementary material.

Supplementary Material 1

Supplementary Material 2

## Data Availability

The original contributions presented in the study are included in the article/supplementary materials, further inquiries can be directed to the corresponding author/s.
